# Recent advances on *Candida albicans* biology and virulence

**DOI:** 10.12688/f1000research.9617.1

**Published:** 2016-10-26

**Authors:** Adnane Sellam, Malcolm Whiteway

**Affiliations:** 1 Infectious Diseases Research Centre-CRI, CHU de Québec Research Center (CHUQ), Université Laval, Quebec City, Quebec, Canada; 2 Department of Microbiology, Infectious Disease and Immunology, Faculty of Medicine, Université Laval, Quebec City, Quebec, Canada; 3 Department of Biology, Concordia University, Montreal, Quebec, Canada

**Keywords:** Candida albicans, antifungal drugs, C. albicans, genome editing, virulence

## Abstract

*Candida albicans* is an important human fungal pathogen, in terms of both its clinical significance and its use as an experimental model for scientific investigation. Although this opportunistic pathogen is a natural component of the human flora, it can cause life-threatening infections in immunosuppressed patients. There are currently a limited number of antifungal molecules and drug targets, and increasing resistance to the front-line therapeutics, demonstrating a clear need for new antifungal drugs. Understanding the biology of this pathogen is an important prerequisite for identifying new drug targets for antifungal therapeutics. In this review, we highlight some recent developments that help us to understand how virulence traits are regulated at the molecular level, in addition to technical advances that improve the ability of genome editing in *C. albicans*.

## Introduction

Fungal diseases now represent global challenges. For example, various crops ^1, 2^, certain bats ^3^, and many amphibians ^4^ have recently come under severe and potentially existential stress due to attacks from fungal pathogens. The impact of fungal disease on human health has also been increasing, in particular because of the growing number of immunocompromised patients resulting from the AIDS epidemic, increased organ transplantation and cancer chemotherapies, and widespread antibiotic use that impacts on the human microbiome. *Candida* species, in particular *Candida albicans*, represent a major component of the disease burden caused by fungi and are the fourth most common cause of nosocomial infections in North American hospitals ^5^. This has led to increasing interest in the biology of *C. albicans* and a continuing improvement in the tools involved in studying this opportunistic pathogen. Because *C. albicans* is an ascomycete, a framework for understanding its biology has been provided by the model ascomycete *Saccharomyces cerevisiae*, arguably one of the best studied and understood eukaryotic organisms. However, much of our recent improvement in understanding the functioning of *C. albicans* has come from identifying the myriad ways in which these two fungi differ.

## Regulatory circuits take the stage

Recent progress has been made in elucidating circuits in *C. albicans* that regulate processes directly implicated in virulence, such as biofilm formation, stress response, and metabolic adaptation. Response to stress is a critical function for an opportunistic pathogen such as *C. albicans* because it has to be able to overcome host defenses to be virulent. Recent evidence points out the role of the ubiquitous heat shock response on cellular processes linked to virulence ^6^. This includes Hsf1 and Hsp90, the heat shock transcription factor and a key heat shock chaperonin, respectively, coordinating chromatin architecture and stress response gene expression to permit adaptation to the mammalian host and potentially the host fever response ^7^. In addition, the heat shock regulatory network connects to key metabolic pathways such as the synthesis of cell membrane components and lipid kinase signaling, all of which are ultimately linked to the cellular response to stress ^8^. These observations provide an overview of just how complex and interrelated are the processes connected to stress response.

Biofilm formation represents a central component of the pathogenicity of *C. albicans*, as the formation of biofilms on medical devices such as catheters contributes significantly to *C. albicans*’ success as a nosocomial pathogen ^9^. Biofilm formation is a complex process, involving multiple cell types and stages. Recently, a framework transcriptional regulatory network has been identified for biofilm formation that includes several transcription factors and interlocking circuits of control ^10^. Investigations into the temporal regulation of biofilm formation has expanded this initial circuit ^11^, showing that not only are the transcriptional controls complex but also they modify over time as the biofilm progresses from an early stage form to maturity. However, transcriptional regulation is not the only process contributing to biofilm formation regulation. The transcriptional circuitry may be connected to the Hsp90 network already implicated in multiple cellular processes ^12^. Additionally, post-transcriptional processes controlling RNA stability through the RNA-binding protein Puf3 and the RNA deadenylase Ccr4 have been found to play a role in regulating matrix production and in connecting biofilm formation to mitochondrial function ^13^.

Metabolic adaptation also plays a key role in the ability of *C. albicans* to colonize different regions of the human body and to survive interactions with the host immune system. While transcriptional regulation is important for this process, post-translational circuitry has also been found to be critical. Recent evidence on the ubiquitination-controlled degradation of enzymes involved in carbohydrate metabolism has established that differences in the enzymes targeted for turnover play a critical role in distinguishing how *S. cerevisiae* and *C. albicans* process sugar ^14^. These data suggest that *C. albicans* is much more flexible in its use of carbon sources and that this flexibility allows the pathogen to exploit varied niches within the mammalian host.

One intriguing element of the role of metabolic adaptation has been the recent identification of transcriptional rewiring events – clear examples of orthologous transcription factors recognizing the same DNA-binding sequences but directing distinct metabolic processes. This provides a clear mechanism for the rapid evolution of circuits that could allow a commensal to adapt to the host, but in general the driving force behind the switches (for example Gal4 regulating glucose metabolism in *C. albicans* but the Leloir pathway in *S. cerevisiae* ^15^ or Mcm1 controlling large and essentially distinct circuits in *S. cerevisiae*, *Kluyveromyces lactis*, and *C. albicans* ^16^) has been unclear. Recent evidence that the rewiring of Ppr1 from control of purine catabolism in *C. albicans* to control of pyrimidine biosynthesis in *S. cerevisiae* could be explained by the evolutionary transition among the *Saccharomycetaceae* to exploit oxygen-poor environments by reducing the presence of oxygen-requiring enzymatic reactions suggests that this rewiring may be generally idiosyncratic, driven by unique responses to metabolic needs and other challenges encountered by the evolving species ^17^. At present, researchers are in the data-collecting mode – we need a more comprehensive picture of circuits and circuit rewiring before we will be in a position to identify and understand the forces driving circuit switches. A combination of location profiling plus expression profiling of activated transcription factors in a limited number of model ascomycetes, coupled with bioinformatic analysis of transcription factor binding motifs across the many sequenced ascomycete lineages, has the potential to map out circuits and circuit evolution. This background will be critical to determining the processes that modulate function and direct species towards benign or pathogenic lifestyles.

An important outcome of all these studies is the recognition that the simple comparison of genomic constitutions is insufficient to establish the behavioral characteristics of even quite closely related organisms. Although model organisms offer important frameworks providing overall understanding of biological functions, individual idiosyncrasies of organisms can preclude the use of models to direct therapeutic interventions to combat pathogens. To rephrase the claim that the proper study of mankind is man, we can say the proper molecular study of pathogenesis is the pathogen.

## Candidalysin, a novel “lytic” virulence factor

Although *C. albicans* has been extensively investigated as a pathogen, only recently has an element that would fit a classical definition of a virulence factor been identified in the fungus. Such factors have been extensively characterized in bacterial pathogens, where their primary (or exclusive) function is to attack or subvert the cells of the host organism. While functions such as the yeast–hyphal transition and the ability to form biofilms are important for *C. albicans* virulence, these characteristics are not just used to target host cells. The recently identified peptide toxin derived from the product of the *ECE1* gene, termed candidalysin, fits the definition of a virulence factor ^18^ in that the peptide serves to target and damage the host cell membrane. Cells that lack *ECE1* can form hyphae and bind to epithelial cells, but they do not trigger membrane damage or induce the MAPK-mediated danger response in these cells. Candidalysin results from the Kex2/Kex1 proteolytic processing of the Ece1 protein and represents only one of eight potential Kex2-generated peptides from this protein. This opens up some questions – what (if anything) are the roles of the other peptides derived from Ece1, and how widespread is the potential for fungal lysins produced from other species? Previous examples of multiple peptides derived from a single protein by Kex2-like enzyme processing (multiple copies of alpha-factor in yeast ^19^) or distinct peptides from pro-opiomelanocortin (POMC) ^20^ suggest that it is likely that the other derived peptides will play functional roles. An extensive survey of *ece1* null mutant phenotypes could provide a place to start looking for such possible roles. The question of similar toxins in other species is equally challenging. While clear Ece1 orthologs would be expected to generate candidalysin-like peptides, cell-membrane-perturbing peptides come in a wide range of sizes and sequences, and identifying possible candidates embedded in larger proteins is a daunting task ^21^. It is perhaps more likely that candidates will be identified by functional assays (as was *ECE1*) than by bioinformatics-based searches for additional lytic peptides in *C. albicans* or in other fungal pathogens. In any event, the search for additional candidates will have been given a strong boost by the clear role of candidalysin in the virulence of *C. albicans*.

## Additional cell types enter the scene

The ability of *C. albicans* to reversibly switch among budding, pseudohyphal growth, or true hyphal growth is seen as being central to its ability to adapt and persist in different niches inside its host. *C. albicans* is also known to form both chlamydospores, a stable resting cell, and opaque cells, which represent the mating-competent state of the organism. This already large repertoire of diversified morphologies was increased recently by the identification of further cell types, highlighting the impressive plasticity and the complexity of this yeast. Pande *et al.* ^22^ demonstrated that a fraction of *C. albicans* cells underwent a developmental switch, driven by the transcription factor Wor1, when passing through the mouse gut. The resulting cells, named GUT (gastrointestinally induced transition) cells, are somewhat similar morphologically to opaque cells; however, they are unresponsive to pheromone and they have few or no pimple structures, which are a defining feature of the mating-competent opaque cells. At the molecular level, GUT cells exhibited a distinct and gut-optimized transcriptional program where genes related to glucose catabolism and iron uptake were repressed while transcripts related to the catabolism of fatty acids and *N*-acetylglucosamine were activated, which fits with the nutrient conditions in the gut where *C. albicans* is opting for commensal growth. This work defined, for the first time, a *C. albicans* specialized commensal cell type. Importantly, this study emphasizes evidence that *C. albicans*-mediated infections are not strictly related to impaired host immunity but are also related to a cell deterministic identity control of commensal–pathogen transitions.

The transition from white to opaque cells, which represents the sexual-competent form, is a well-known phenomenon in *C. albicans* ^23^. In terms of fitness inside the host, white cells are more efficient in causing systemic candidiasis while opaque cells are better at mediating cutaneous infections ^24^. Tao *et al.* ^25^ recently uncovered a novel intermediate phase between the white and opaque phenotypes called the gray phenotype. The three cell states form a robust tri-stable white–gray–opaque phenotypic switching system under the coordinated control of the two transcription factors Wor1 and Efg1. The gray cells are similar to opaque cells in general shape; however, they exhibit small size and low mating efficiency. The gray cell type has unique virulence characteristics, with a high ability to cause cutaneous infections and a reduced capacity in colonizing internal organs such as the kidney, lung, and brain ^25^. A similar gray state was also observed in *Candida tropicalis,* a yeast closely related to *C. albicans* ^26^.

While GUT and gray cell types are determined by genetically dynamic transcriptional regulatory circuits, a new morphology termed “Trimeras” (three connected cells composed of a mother, daughter, and granddaughter bud) was identified as being determined by the ploidy of *C. albicans* cells ^27^. Trimeras were formed as a consequence of exposure to the widely used antifungal fluconazole or to other related azoles. The induced altered ploidy (aneuploidy), which is the consequence of unequal chromosome segregation in the progeny of Trimera cells, might consequently occur as a resistance mechanism, as was previously shown by the same group ^28^.

## Tailored CRISPR-Cas9 genome editing tools for *C. albicans*

The CRISPR-Cas (clustered regularly interspaced short palindromic repeats and CRISPR-associated) genome editing tool is a revolutionizing technology. The tool has been derived from a bacterial immune system that provides defense against repeated phage attacks ^29^ and has been engineered to mediate targeted double-strand breaks (DSBs) in the genome of different eukaryotic organisms, including *C. albicans* ^30, 31^. The principle of CRISPR-Cas9 is based on the observation that the bacterial endonuclease Cas9 is guided by an RNA (gRNA) determining the site specificity of the DNA-cutting activity. CRISPR tools for *C. albicans* have been developed by Gerald Fink’s ^30^ and Aaron Mitchell’s ^31^ groups to mediate gene deletion. Vyas *et al.* ^30^ provided a CRISPR toolbox where a *C. albicans* codon-optimized Cas9 nuclease and gRNA are both stably integrated in distinct chromosomal locations in the genome. *C. albicans* is a diploid organism, and this system permits an efficient simultaneous mutagenesis of the two alleles of each targeted gene, a big improvement on the classical two-step cassette deletion methodology ^32^. Vyas *et al.* had shown that the source of mutation in *C. albicans* using the CRISPR system could be from erroneous DSB repair or a donor DNA (also referred to as a repair template). The *Candida* CRISPR-Cas system also allowed mutagenesis of multiple genes, gene families, and essential genes ^30^. Recently, the Mitchell group has adapted this CRISPR-Cas9 tool and offered an alternative CRISPR system where the Cas9 and gRNA cassettes can function transiently without being integrated in the genome ^31^.

Although haploid strains of *C. albicans* have been identified ^33^, they have proven to be quite unstable, and the typical diploid nature of *C. albicans* has made the construction of large-scale disruption collections a technical challenge ^34^. However, the potential for targeting all *C. albicans* ORFs using CRISPR-Cas9 is evident; in the longer term, we expect that genome-wide homozygous deletion collections will be made available to the research community through this methodology. This will lead to a more comprehensive knowledge of the biology of this opportunistic yeast and a better understanding of relevant virulence traits which so far have been limited to the use of a lower scale of generalist ^35^ or specialized ^36– 38^ mutant collections. However, we are only beginning to witness the potential of CRISPR-Cas9 in uncovering the function of *C. albicans* genes and their roles in controlling relevant biological traits. In addition to targeted gene editing, recent studies revealed that CRISPR-Cas9 is a versatile tool for promoter-mediated transcriptional control ^39^ in addition to utility in epigenetic modification ^40^ and genome imaging ^41^. In this regard, this recent adaptation of CRISPR tools can be used in *C. albicans* to improve our understanding of genetic circuits that control cell types, drug resistance mechanisms, stress responses, metabolic adaptation, and other different virulence traits.

## Outlook

In recent years, impressive advances have been made in the *C. albicans* field, making it the currently best-studied fungal pathogen. Investigations have yielded unprecedented insight into the regulatory circuits controlling different virulence functions as well as the commensal state. However, there is a clear need to have the types of genome-wide deletion and overexpressing mutant collections in addition to epitope- or fluorescent protein-tagged genes that have been developed in *S. cerevisiae*. The availability of such reagents in this opportunistic pathogenic yeast will allow unbiased comparisons, at the genome scale, of how conserved the functions of genes/phenomes, transcriptional and signaling pathways, and protein complexes are compared to those of the saprophytic yeast *S. cerevisiae*. This will sketch a comprehensive regulatory model that will lead to an improved understanding of how biological circuits evolve and rewire as a result of lifestyle variations and should ultimately improve our ability to target new therapeutic approaches to treat *C. albicans* infections.

## Abbreviations

Cas9, CRISPR-associated protein 9; CRISPR, clustered regularly interspaced short palindromic repeats; DSB, double-strand breaks; gRNA, guide RNA; GUT, gastrointestinally induced transition.
